# ANKS1B is a smoking-related molecular alteration in clear cell renal cell carcinoma

**DOI:** 10.1186/1471-2490-14-14

**Published:** 2014-01-31

**Authors:** Jeanette E Eckel-Passow, Daniel J Serie, Brian M Bot, Richard W Joseph, John C Cheville, Alexander S Parker

**Affiliations:** 1 Division of Biomedical Statistics and Informatics, Mayo Clinic, Rochester, MN, USA; 2 Department of Health Sciences Research, Mayo Clinic, 4500 San Pablo Road, Jacksonville, FL 32224, USA; 3 Statistical Genetics, Sage Bionetworks, Seattle, WA, USA; 4 Department of Hematology and Oncology, Mayo Clinic, Jacksonville, FL, USA; 5 Laboratory Medicine and Pathology, Mayo Clinic, Rochester, MN, USA

## Abstract

**Background:**

An association between cigarette smoking and increased risk of clear cell renal cell carcinoma (ccRCC) has been established; however, there are limited data regarding the molecular mechanisms that underlie this association. We used a multi-stage design to identify and validate genes that are associated with smoking-related ccRCC.

**Methods:**

We first conducted a microarray study to compare gene expression patterns in patient-matched ccRCC and normal kidney tissues between patients with (n = 23) and without (n = 42) a history of smoking. Analyses were first stratified on obesity status (the other primary risk factor for ccRCC) and then combined and analyzed together. To identify genes where the fold change in smokers relative to non-smokers was different in tumor tissues in comparison to patient-matched normal kidney tissues, we identified Affymetrix probesets that had a significant tissue type-by-smoking status interaction pvalue. We then performed RT-PCR validation on the top eight candidate genes in an independent sample of 28 smokers and 54 non-smokers.

**Results:**

We identified 15 probesets that mapped to eight genes that had candidate associations with smoking-related ccRCC: ANKS1B, ACOT6, PPWD1, EYS, LIMCH1, CHRNA6, MT1G, and ZNF600. Using RT-PCR, we validated that expression of ANKS1B is preferentially down-regulated in smoking-related ccRCC.

**Conclusion:**

We provide the first evidence that ANKS1B expression is down regulated in ccRCC tumors relative to patient-matched normal kidney tissue in smokers. Thus, ANKS1B should be explored further as a novel avenue for early detection as well as prevention of ccRCC in smokers.

## Background

Currently, cigarette smoking is an established risk factor for the development of clear cell renal cell carcinoma (ccRCC) [1]. Indeed, authors of a meta-analysis involving 26 epidemiologic studies spanning 37 years concluded that the risk of ccRCC among ever smokers is approximately 40% higher compared to lifetime never smokers [2]. From a population-based perspective, previous investigators have suggested that cigarette smoking alone accounts for approximately 20-25% of the ccRCCs diagnosed in the U.S. [3,4]. While smoking is an established risk factor for ccRCC, what remain unclear are the specific somatic molecular alterations that underlie this well-reported association. Identification of specific alterations at the cellular level that link smoking to ccRCC development has the potential to further solidify a causal association, advance our understanding of the etiology of this disease and possibly extend even further into more focused measures of early detection and prevention.

To address the need to better understand the molecular underpinnings of smoking-related ccRCC, we sought to identify candidate genes that are differentially expressed in ccRCC tumors that develop in smokers compared to non-smokers. Thus, we employed the Affymetrix U133 Plus 2.0 platform to compare somatic gene expression profiles between patient-matched ccRCC and normal kidney tissues from patients with and without a history of smoking, controlling for obesity status (the other primary risk factor for ccRCC [4,5]). Although other risk factors have been reported in the literature, smoking and obesity are the only epidemiological risk factors that have been consistently validated as increasing risk of ccRCC. Following our microarray-based discovery efforts, we then validated our top candidate genes by employing RT-PCR on an independent set of ccRCC and patient-matched normal kidney tissue samples from smokers and non-smokers. Using this multi-stage design, we report that ANKS1B is a smoking-related alteration in ccRCC.

## Methods

### Ethics statement

This study was approved by the Mayo Clinic Institutional Review Board. All participants provided written consent to participate in this study.

### Overview

For this investigation, we employed a multi-stage design that allowed us to take into account potential confounding effects of obesity, the other consistently-reported risk factor of ccRCC [5], and seek validation of our top candidate genes. Briefly, in stage 1 we only considered non-obese subjects and used the Affymetrix platform on patient-matched ccRCC and normal kidney tissues from smokers and non-smokers to identify candidate smoking-related gene expression changes in ccRCC. In stage 2, we again used the Affymetrix platform on patient-matched ccRCC and normal kidney tissues from smokers and non-smokers; however, this time we included only obese subjects. That is, we aimed to identify smoking-related genes that were not dependent on obesity status. With the list of candidate genes narrowed down, in stage 3 we performed RT-PCR validation on the top candidates in an independent set of patient-matched ccRCC and normal kidney tissues. We provide more detail on the design and selection of the subjects for each stage in the sections below.

### Patient selection

#### ***Stage 1: Affymetrix microarrays on non-obese ccRCC subjects***

The objective of stage 1 was to perform a genome-wide scan and identify candidate genes that are associated with smoking-related ccRCC. To do so, we compared gene expression between patients with and without a history of smoking across patient-matched tumor and normal kidney samples. Upon approval from our Institutional Review Board, we identified patients treated with radical nephrectomy or nephron-sparing surgery for unilateral, sporadic ccRCC between 2000 and 2006 from our Nephrectomy Registry. We then excluded all patients with a body mass index (BMI) > 30 kg/m^2^ as well as patients with late stage tumors (pT4) and patients with high-grade tumors (grade 4). The decision to remove patients with a BMI > 30 in stage 1 was based on the fact that obesity represents the only other widely reproducible risk factor for ccRCC development and thus we wanted to match by obesity status. The removal of late-stage and high-grade subjects was based on our desire in stage 1 to identify changes that occur early in ccRCC carcinogenesis. Based on these criteria, we identified 46 non-obese subjects that had both fresh-frozen normal kidney and tumor tissue available for study; 16 of which had a history of smoking and 30 had no history of smoking. We obtained smoking data from risk factor questionnaires completed at time of surgery and from medical chart review where necessary. Using these data, we defined non-smokers as anyone who reported never smoking cigarettes on the questionnaire or to their physician during a standard patient history taken prior to surgery. For smokers, we required that the subject report greater than 20 pack-years of smoking on either the questionnaire or during the patient history.

#### ***Stage 2: Affymetrix microarrays on obese ccRCC subjects***

As noted above, because obesity is the other widely acceptable risk factor and we wanted to identify molecular markers that were not dependent on obesity status, we performed a two-stage design stratifying by obesity status. Thus, we repeated our design and analysis from stage 1 but this time we only used obese subjects. Our rationale for this second stage of discovery is that by moving into an obese population we would have the opportunity to further screen the candidates from stage 1 by looking for genes that still have a smoking-related expression signal even among subjects with another primary risk factor for ccRCC. The subjects in stage 2 were similar to stage 1 (i.e. unilateral, sporadic, pT stage 1-3, grade 1-3) with the exception that they all had a BMI > 30 kg/m^2^ at time of surgery. As such, stage 2 consisted of 19 obese ccRCC subjects that had both fresh-frozen normal kidney and tumor tissue available for study; 7 of which had a history of smoking and 12 had no history of smoking. We used the same criteria to define smokers and non-smokers as described above for stage 1.

#### ***Stage 3: RT-PCR validation on non-obese ccRCC subjects***

With our discovery-based steps complete, the objective of stage 3 was to seek independent validation of the candidate genes we identified in stages 1-2. The patients in stage 3 consisted of 82 non-obese patients that had both fresh-frozen normal kidney and tumor tissue available for study; 28 of which had a history of smoking and 54 had no history of smoking. For this important validation step we moved back into the setting of only evaluating non-obese patients to allow for the most robust chance of validation. For this validation stage, we used the same criteria to define smokers and non-smokers as described above for stage 1.

### Tissue preparation and laboratory assays

#### ***Tissue samples***

An experienced urologic pathologist identified fresh-frozen blocks with representative tumor and normal kidney tissue for each patient involved in stages 1-3. For those patients with a ccRCC tumor that showed mixed grade, the study pathologist selected the block with the highest grade tumor for dissection. After the appropriate blocks were selected, a histotechnologist macrodissected two five-micron sections from each of the fresh-frozen tumor and corresponding normal kidney tissue blocks. The Mayo Biospecimen Accessioning and Processing Core performed RNA extractions using kits and protocols from the Qiagen miRNEasy kit and Qiagen Qiacube instrument. The RNA was DNAse treated on the column prior to elution. We assessed RNA quantity and quality using Nanodrop Spectrophotometer and Agilent.

#### ***Affymetrix microarrays***

Microarray analysis was conducted according to manufacturer’s instructions for the Affymetrix One Cycle Target Labeling and Control Reagents kit (Santa Clara, CA). Briefly, cDNA was generated from five micrograms of total RNA using SuperScript II reverse transcriptase (Invitrogen, Carlsbad, CA) and T7 Oligo(dT) primer. Subsequently, the products were column-purified (Affymetrix) and then *in vitro* transcribed to generate biotin-labeled cRNA. The IVT products were then column-purified, fragmented, and hybridized onto Affymetrix U133 Plus 2.0 GeneChips® at 45°C for 16 h. Subsequent to hybridization, the arrays were washed and stained with streptavidin-phycoerythrin, then scanned in an Affymetrix GeneChip® Scanner 3000 (Santa Clara, CA). All control parameters were confirmed to be within normal ranges before normalization was initiated. The data discussed in this publication have been deposited in NCBI’s Gene Expression Omnibus and are accessible through GEO Series accession number GSE46699 (http://www.ncbi.nlm.nih.gov/geo/query/acc.cgi?acc=GSE46699).

#### ***Microarray data normalization and statistical methods***

The data used herein are comprised of two batches of samples that were processed at two different time periods (see Supplementary Methods in [6]). Base-2 logarithm transformed intensity data from the two batches of samples were normalized within each batch using frozen robust multi-array analysis (frozen RMA) [7]. Frozen RMA was specifically designed to preprocess arrays in batches and subsequently allow the data to be combined for downstream analyses.

**Figure 1:**
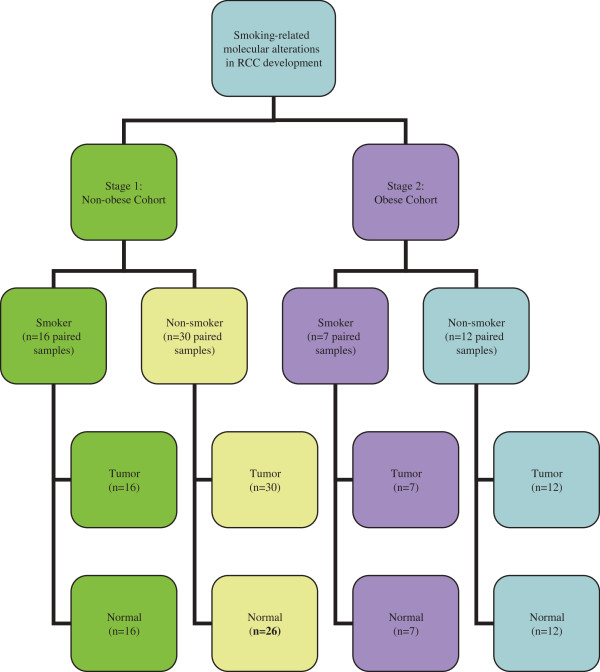
**Experimental design for stage 1 and stage 2.** *Normal tissue did not pass RNA or microarray quality-control metrics for 4 normal tissue samples.

The samples used in stage 1 and stage 2 are shown in Figure 1. Stage 1 and stage 2 data were analyzed separately and then combined and analyzed as a whole. Linear mixed models were fit to the normalized intensity data for each probeset. Within the linear mixed model, tissue type (tumor/normal), smoking status (smoker/non-smoker) and a smoking status-by-tissue type interaction were included as fixed effects while a random intercept was fit on a per patient basis to account for the patient-matched tumor and normal samples. The smoking status-by-tissue type interaction was included to identify probesets where the fold change between smokers versus non-smokers was different in tumor in comparison to normal tissue. Probesets with a smoking status-by-tissue type interaction p-value <0.01 in stage 1 were identified as having a potential association with smoking-specific alterations in ccRCC and therefore were determined to be good candidates for further evaluation in stage 2. We acknowledge that this p-value threshold does not account for multiple testing at the conservative Bonferroni level. However, probesets that are consistently identified in stage 1 at this nominal significance and subsequently in stage 2 with a smoking status-by-tissue type interaction pvalue <0.05 and then maintained a smoking status-by-tissue type interaction p-value <0.01 in the analysis of the combined data were deemed to be good candidates for further validation in stage 3. To determine how the fold change differed across tumor and normal specimens, we also calculated the fold change of normalized expression for smokers versus non-smokers in normal tissue as well as the fold change of smokers versus non-smokers tumor tissue. All statistical tests were performed using a Linux release of R version 2.14. All probeset-to-gene mapping was done using the hgu133plus2.db (version 2.9.0).

#### ***Gene expression by fluidigm quantitative PCR***

Samples were reverse transcribed according to the manufacturer’s instructions for the High Capacity Reverse Transcription kit (Applied Biosystems, Foster City, CA). Briefly, 50 ng of total RNA was reverse transcribed in a 20 μl reaction mixture containing 0.8 μl of 100 nM dNTP, 2.0 μl RT buffer, 1.0 μl of reverse transcriptase (50U/μl), 2 μl of RT primer. The reaction mixture was mixed and incubated as follows; 25°C for 10 min, 37°C for 2 h, and then 85°C for 5 min, followed by a 4°C hold. Pre-amplification of cDNA was initiated by creating a pool of 24 TaqMan mRNA Assays at a final concentration of 0.2X for each assay. The pre-PCR amplification reaction was then performed in a 5 μl reaction mixture containing 2.5 μl TaqMan PreAmp Master Mix (2X), 1.25 μl of 24-pooled TaqMan assay mix (0.2X) and 1.25 μl of cDNA. The pre-amplification PCR was performed according to the following cycling conditions: one cycle 95°C for 10 min, 14 cycles at 95°C for 15 sec and then 60°C for 4 min. After pre-amplification PCR, the product was diluted 1:5 with dH_2_O and stored at -20°C until needed for amplification.

Quantitative PCR of the mRNA targets was carried out using the 48.48 dynamic array (Fluidigm, South San Francisco, CA) following the manufacturer’s protocol. Briefly, a 5 μl sample mixture was prepared for each sample containing 2x TaqMan Universal Master Mix (with UNG), 20X GE Sample Loading Reagent and each of diluted pre-amplified cDNA. Five microliters of Assay mix was prepared with one 20X TaqMan mRNA assay (final concentration 10x) and 2X Assay Loading Reagent. The dynamic array was primed with control line fluid in the IFC controller and samples and assay mixes was loaded into the appropriate inlets. The chip was then returned to the IFC controller for loading and mixing, and then placed in the BioMark Instrument for PCR at 50°C for 2 min and 95°C for 10 min, followed by 40 cycles at 95°C for 15 sec and 60°C for 1 min. The data were analyzed with the Real-Time PCR Analysis Software (Fluidigm, South San Francisco, CA).

#### RT-PCR data normalization and statistical methods

Normalization was carried out as discussed previously [8]. In brief, the negative CT (denoted hereafter as -CT) values for the two control genes (POLR2A and ACTB) were averaged on a per sample basis and the average was subtracted from the -CT value for each sample. As was done for the Affymetrix microarray data, linear mixed models were fit to the normalized –CT data for each gene. Within the linear mixed model, tissue type (tumor/normal), smoking status (smoker/non-smoker) and a smoking status-by-tissue type interaction were included as fixed effects while a random intercept was fit on a per patient basis.

## Results

### Patient characteristics

We provide a comparison of demographic and clinical characteristics between smokers and non-smokers for the patients in each of the three stages of our study in Table 1. Although in all three stages there was a trend for smokers to more likely be male than the non-smokers, this trend was only statistically significant in stage 3. In contrast, we observed no differences in age categories or in tumor grade between smokers and non-smokers across the three stages. In stage 1, smokers were more likely to have later stage disease compared to non-smokers; however, the stage distribution was similar between smokers and non-smokers among patients in stages 2-3. Finally, across all three stages, there was no significant difference in presence of necrosis in smokers compared to non-smokers.

**Table 1:** Demographic and clinicopathologic characteristics for subjects in each stage of the multi-stage design

	**Affymetrix microarray**						**RT-PCR**		
	**Stage 1: non-obese subjects**			**Stage 2: obese subjects**			**Stage 3: non-obese subjects**		
	**Non-smokers 30 (65%)**	**Smokers 16 (35%)**	**p-value**	**Non-smokers 12 (63%)**	**Smokers 7 (37%)**	**p-value**	**Non-smokers 54 (66%)**	**Smokers 28 (34%)**	**p-value**
Gender			0.2170			0.1698			0.0273
Male	14 (47%)	11 (69%)		4 (33%)	5 (71%)		30 (56%)	23 (82%)	
Female	16 (53%)	5 (31%)		8 (67%)	2 (29%)		24 (44%)	5 (18%)	
Age at surgery			0.2537			0.8584			0.4597
<50	3 (10%)	3 (19%)		2 (17%)	1 (14%)		9 (17%)	5 (18%)	
50-79	24 (80%)	13 (81%)		9 (75%)	6 (86%)		41 (76%)	23 (82%)	
≥80	2 (7%)	0		1 (8%)	0		4 (7%)	0	
Unknown	1 (3%)	0		0	0		0	0	
Nuclear grade			0.2032			0.5834			0.2000
1	3 (10%)	1 (6%)		4 (33%)	2 (29%)		5 (9%)	3 (11%)	
2	18 (60%)	7 (44%)		8 (67%)	4 (57%)		17 (32%)	7 (25%)	
3	9 (30%)	8 (50%)		0	1 (14%)		24 (44%)	11 (39%)	
4	0	0		0	0		8 (15%)	7 (25%)	
Pathologic tumor stage			0.0123			0.5926			0.4713
pT1	24 (80%)	7 (44%)		9 (75%)	6 (86%)		31 (57%)	11 (39%)	
pT2	2 (7%)	2 (12%)		2 (17%)	1 (14%)		4 (7%)	4 (14%)	
pT3	4 (13%)	7 (44%)		1 (8%)	0		18 (33%)	13 (46%)	
pT4	0	0		0	0		1 (2%)	0	
Presence of necrosis			0.2829			1.0000			0.4739
Yes	5 (17%)	5 (31%)		1 (8%)	1 (14%)		19 (35%)	13 (46%)	
No	25 (83%)	11 (69%)		11 (92%)	6 (86%)		34 (63%)	15 (54%)	
Unknown	0	0		0	0		1 (2%)	0	

### Discovery of genes associated with smoking-related ccRCC (stage 1 and stage 2 results)

We identified 305 probesets that had a smoking status-by-tissue type interaction p-value <0.01 in stage 1 (non-obese cohort). Of the 305 probesets we identified in stage 1, 15 also had a smoking status-by-tissue type interaction p-value <0.05 in stage 2 (obese cohort) and maintained a p-value <0.01 in the analysis of the combined data (Table 2). Of these 15 probesets, only nine were mapped to known genes. Due to the fact that the Affymetrix platform contains multiple probesets that map to the same gene, in addition to showing the 15 probesets that met our pre-defined filtering criteria, Additional file 1 provides results for all additional probesets that map to these 9 genes and demonstrates that the fold change estimates are consistent across probesets that map to the same gene. In normal kidney tissue ANKS1B, ACOT6, EYS, CHRNA6, MT1G and UTY were up regulated in smokers in comparison to non-smokers; however, these genes tended to be down regulated in smokers versus non-smokers in ccRCC tumor tissue. Conversely, in normal kidney tissue PPWD1, LUMCH1 and ZNF600 were down regulated in smokers compared to non-smokers; however, these genes were up regulated in smokers versus non-smokers in ccRCC tumor tissue. We selected eight of these nine candidate genes for follow-up validation using RT-PCR in stage 3; we chose not to attempt to validate UTY since it is located on chromosome Y and likely reflects the fact that smokers were more likely to be male than non-smokers.

**Table 2:** Results for stage 1, stage 2 and the combined samples from stage 1 and stage 2

**Smokers vs non-smokers**											
**Affymetrix probeset**	**Gene**	**Chrom**	**Stage 1: non-obese patients**			**Stage 2: obese patients**			**Combined (stage 1 + stage 2)**		
			**Fold change in tumor tissue (p-value)**	**Fold change in normal tissue (p-value)**	**Interaction p-value**	**Fold change in tumor tissue (p-value)**	**Fold change in normal tissue (p-value)**	**Interaction p-value**	**Fold change in tumor tissue (p-value)**	**Fold change in normal tissue (p-value)**	**Interaction p-value**
240292_x_at	ANKS1B	12	0.92 (0.013)	1.08 (0.02)	0.00082	0.98 (0.64)	1.11 (0.005)	0.018	0.94 (0.014)	1.09 (0.0013)	0.00005
241949_at	ACOT6	14	0.88 (0.0098)	1.07 (0.15)	0.00085	0.89 (0.1)	1.04 (0.56)	0.034	0.88 (0.0019)	1.06 (0.13)	0.00006
236999_at	PPWD1	5	1.13 (0.0062)	0.99 (0.74)	0.0015	1.2 (0.0034)	1.04 (0.5)	0.038	1.14 (0.00023)	1.0 (0.99)	0.00016
233996_x_at	EYS	6	0.94 (0.022)	1.03 (0.27)	0.0022	1.0 (1)	1.06 (0.045)	0.046	0.96 (0.033)	1.04 (0.077)	0.00026
241459_at	LIMCH1	4	1.27 (0.06)	0.89 (0.37)	0.0077	1.9 (0.0032)	1.09 (0.65)	0.02	1.43 (0.0011)	0.95 (0.62)	0.00047
207568_at	CHRNA6	8	0.92 (0.24)	1.15 (0.064)	0.0075	0.95 (0.62)	1.23 (0.034)	0.037	0.93 (0.2)	1.17 (0.0069)	0.00058
210472_at	MT1G	16	1.0 (0.97)	1.47 (0.00013)	0.0045	0.93 (0.62)	1.4 (0.032)	0.018	0.98 (0.82)	1.45 (0.0000099)	0.00061
242463_x_at	ZNF600	19	1.48 (0.0064)	0.98 (0.87)	0.0077	1.78 (0.001)	1.31 (0.083)	0.045	1.56 (0.00013)	1.07 (0.53)	0.0012
210322_x_at	UTY	Y	0.98 (0.76)	1.13 (0.079)	0.0086	0.96 (0.72)	1.22 (0.075)	0.0093	0.98 (0.68)	1.16 (0.012)	0.00029
1557478_at	NA	NA	1.22 (0.03)	0.95 (0.55)	0.00052	1.88 (0.00002)	1.31 (0.022)	0.0011	1.38 (0.00012)	1.04 (0.62)	0.000002
1558410_s_at	NA	NA	1.42 (0.013)	0.93 (0.58)	0.0049	2.49 (0.0000087)	1.63 (0.0038)	0.017	1.66 (0.000044)	1.1 (0.44)	0.0006
210717_at	NA	NA	1.74 (0.00062)	0.89 (0.47)	0.0033	2.12 (0.00026)	1.41 (0.053)	0.027	1.83 (0.000011)	1.03 (0.8)	0.00086
232324_x_at	NA	NA	1.17 (0.004)	0.97 (0.52)	0.0081	1.27 (0.0018)	1.03 (0.61)	0.039	1.2 (0.000058)	0.99 (0.77)	0.0011
232369_at	NA	NA	1.36 (0.021)	0.89 (0.4)	0.0081	2.07 (0.00065)	1.3 (0.15)	0.031	1.53 (0.00016)	1.0 (0.99)	0.00078
244290_at	NA	NA	1.32 (0.0038)	0.99 (0.94)	0.0015	1.66 (0.000041)	1.26 (0.022)	0.033	1.4 (0.000035)	1.06 (0.42)	0.00019

The smoking status-by-tissue type interaction p-value is provided as well as the fold change of expression in smokers relative to non-smokers and corresponding p-values.

Chrom denotes chromosome.

NA denotes that there is no gene annotation available for the corresponding Affymetrix probeset.

#### ***Independent RT-PCR validation (stage 3 results)***

Of the eight genes interrogated via RT-PCR in stage 3, only ANKS1B validated as having an expression pattern that was consistent with what was observed in stages 1 and 2 (Table 3). Specifically, in stage 1 (non-obese cohort) ANKS1B had a tissue type-by-smoking status interaction p-value of 0.0008; the fold change of expression between smokers and non-smokers was 1.08 (p = 0.02) in normal tissues and 0.92 (p = 0.01) in tumor tissues (Table 2). These results were consistent in stage 2 (obese cohort) with an interaction p-value of 0.018 and a fold change of expression between smokers and non-smokers of 1.11 (p = 0.005) in normal tissue and 0.98 (p = 0.64) in tumor tissue (Table 2). Furthermore, the additional 4 probesets that map to ANKS1B showed similar fold change estimates as the proband probeset that met our pre-defined filtering criteria (Additional file 1). Performing RT-PCR on an independent cohort of 82 non-obese subjects (stage 3), we validated these results with an interaction p-value of 0.0051; the fold change of expression between smokers and non-smokers was 1.35 (p = 0.06) in normal tissues and 0.95 (p = 0.76) in tumor tissues.

**Table 3:** RT-PCR results for stage 3

**Gene**	**Chrom**	**Stage 3: non-obese patients**		
		**Fold change in tumor tissue (p-value)**	**Fold change in normal tissue (p-value)**	**Interaction p-value**
ANKS1B	12	0.95 (0.76)	1.35 (0.06)	0.0051
ACOT6	14	1.21 (0.53)	1.03 (0.92)	0.56
PPWD1	5	0.97 (0.66)	0.94 (0.44)	0.67
LIMCH1	4	1.04 (0.81)	1.05 (0.76)	0.93
CHRNA6	8	0.92 (0.71)	0.63 (0.038)	0.095
MT1G	16	1.15 (0.72)	1.27 (0.55)	0.80
ZNF600	19	1.05 (0.62)	1.00 (0.99)	0.52

The smoking status-by-tissue type interaction p-value is provided as are the fold change of expression in smokers relative to non-smokers and corresponding p-values.

Chrom denotes chromosome.

## Discussion

Based on the current literature, there is little question regarding the role of cigarette smoking in the etiology of ccRCC; however, what remains unclear is exactly how smoking acts within the body (specifically within the kidney itself) to increase a person’s risk of developing ccRCC. Related to this, tobacco smoke contains a vast number of chemicals, with about 50 of those chemicals being classified as human carcinogens [9]. Inhaled chemical carcinogens from cigarette smoke, like any other chemical that enters the human body, are subject to extensive metabolism. The majority of this metabolism is directed toward deactivation of the particular chemical and eventual excretion. However, an important fraction of the metabolic process results in the conversion of the ingested compound to highly reactive metabolite(s) that possess the ability to bind to intercellular components (i.e. DNA) and induce changes in their structure; changes that may or may not lead to the transformation of normal cells to tumor cells. Given that the kidney is the main filtration organ of the blood and is known to locally produce enzymes involved in xenobiotic metabolism, it is theorized to be at high exposure to any smoking-related carcinogen. In fact, researchers have reported that the urine of smokers has increased mutagenic activity compared to non-smokers [10]. While this primary theory of how smoking increases the risk of ccRCC does exist, little progress has been made towards illuminating the actual molecular target(s) that are altered by smoking carcinogens in the development of ccRCC.

ANKS1B, Ankyrin repeat and sterile alpha motif domain-containing protein 1B, is a tyrosine kinase signal transduction gene that is primarily expressed in the brain and testis. Here, we demonstrate for the first time that expression of the ANKS1B gene is associated with smoking-related ccRCC development. ANKS1B is involved in apoptosis and thus has the potential to play a key role in cancer development [11]. From our observational data, we show that ANKS1B is up regulated in smokers relative to non-smokers in normal kidney tissue; however, it is down regulated in smokers relative to non-smokers in ccRCC tumor tissue. Thus, ANKS1B expression in smokers is down regulated in the tumor tissue in comparison to the patient-matched normal kidney tissue and this down regulation is potentially a key event that supports ccRCC development. Interestingly, Lin et al. [12] recently evaluated the association of germline SNPs within apoptotic pathway genes with lung cancer risk – in which smoking is also a major risk factor – and identified 2 SNPs in ANKS1B (rs1549102 and rs11110099) that had statistically significant associations. What remains unclear is whether these SNPs are also found in lung cancer tissues and whether they are functionally associated with expression or activity of the ANKS1B protein. That notwithstanding, these results from another smoking-related cancer further suggest a possible role for ANKS1B to be a smoking-related molecular alteration in cancer and underscore the potential for these results to advance the knowledge of ccRCC etiology and prevention. Indeed, in addition to advancing our understanding of the pathways involved in smoking-related ccRCC, alterations in ANKS1B could also potentially be used for early detection and prevention in smokers. That being said, we acknowledge that our findings must first be validated at the protein level. Moreover, there is a need to link alterations in ANKS1B to smoking-related ccRCC in a more robust epidemiologic study design. Particularly, using a larger case–control study or a large prospective-cohort study where it would be feasible to adjust for additional reported risk factors, to study the dose–response relationship of smoking with ANKS1B and lastly, to study the association of smoking with molecularly-defined ccRCC subtypes.

We used a discovery-based approach to identify smoking-specific molecular alterations associated with ccRCC development that can be followed up in more focused investigations. Having said that, the key limitations of our approach include our focus on expression changes at the RNA level (compared to protein expression or alterations at DNA level) and our overall limited generalizability (tertiary referral center, >95% of patients are Caucasian). We acknowledge that our cohort has differences between the ccRCC tumors in the smokers and non-smokers that were studied. First, smokers were more likely to be male than non-smokers. Additionally, smokers in our study were more likely to have later stage disease compared to non-smokers in stage 1; however, the stage distributions were similar between smokers and non-smokers in stages 2-3. Since ANKS1B showed similar results in all 3 stages it is likely not simply a marker associated with later-stage disease. With those limitations in mind, the specific strengths of our design include the use of only clear cell RCC subtype (the most common histologic subtype), exclusion of late stage and high grade tumors in the discovery stages (to focus on events linked to early ccRCC development), use of packyears > 20 years to define smokers (those at theorized high exposure to smoking carcinogens) and access to data on obesity in order to account for the other primary risk factor for ccRCC.

Our study was designed specifically to identify smoking-related molecular alterations that are associated with ccRCC development. As a result, we evaluated patient-matched tumor and normal kidney samples from both smokers and non-smokers. Thus, our potential targets of interest were those that had a statistically significant smoking status-by-tissue type interaction. It is worth noting that if cancer is not of interest and future investigators are interested in simply identifying genes that are associated with only smoking, our publicly available data could be further explored to identify genes with a significant smoking main effect.

## Conclusion

In summary, we demonstrated that ANKS1B expression is associated with smoking-related ccRCC. Interestingly, ANKS1B was recently shown to be associated with cancer by Lin et al. [12], where they showed that 2 SNPs in ANKS1B are associated with risk of lung cancer. Here, we showed that ANKS1B is under expressed in ccRCC tumor tissue in comparison to patient-matched normal. Given the role of ANKS1B as an enhancer of apoptosis, down regulation of this gene could be involved in increasing the risk of ccRCC development.

## Availability of supporting data

The data supporting the results of this article are available in the Gene Expression Omnibus repository and are accessible through GEO Series accession number GSE46699 [http://www.ncbi.nlm.nih.gov/geo/query/acc.cgi?acc=GSE46699].

## Competing interests

The authors declare that they have no competing interests.

## Authors’ contributions

JEP participated in the conception and design, assisted in the statistical analyses and drafted the manuscript. DJS performed the statistical analyses and helped draft the manuscript. BMB participated in the conception and design, performed the statistical analyses and revised the manuscript. RWJ participated in the interpretation and revised the manuscript. JCC reviewed the pathology of all subjects and revised the manuscript. ASP conceived the study, participated in the interpretation, drafting and final approval of the manuscript. All authors read and approved the final manuscript.

## Pre-publication history

The pre-publication history for this paper can be accessed here:

http://www.biomedcentral.com/1471-2490/14/14/prepub

## Supplementary Material

Additional file 1 **Results for stage 1, stage 2 and the combined samples from stage 1 and stage 2.** Fold change of expression in smokers relative to non-smokers and p-values are provided. The proband probesets that met our filtering criteria are in bold font; the results for all additional probesets that map to the same gene are provided to demonstrate consistency of results across probesets targeting the same gene. Click here for file
