# Supplementary material for: ANKS1B is a smoking-related molecular alteration in clear cell renal cell carcinoma
Source: BMC Urol. 2014 Jan 31;14:14. doi: 10.1186/1471-2490-14-14 (PMC3944917; doi:10.1186/1471-2490-14-14)
Supplement: Additional file 1 — Results for stage 1, stage 2 and the combined samples from stage 1 and stage 2. Fold change of expression in smokers relative to non-smokers and p-values are provided. The proband probesets that met our filtering criteria are in bold font; the results for all additional probesets that map to the same gene are provided to demonstrate consistency of results across probesets targeting the same gene. [file 1471-2490-14-14-S1.docx]

**Additional Table 1:** Results for stage 1, stage 2 and the combined samples from stage 1 and stage 2. Fold change of expression in smokers relative to non-smokers and p-values are provided. The proband probesets that met our filtering criteria are in bold font; the results for all additional probesets that map to the same gene are provided to demonstrate consistency of results across probesets targeting the same gene.

| Smokers vs Non-Smokers | | | | | | | | | | | |
| --- | --- | --- | --- | --- | --- | --- | --- | --- | --- | --- | --- |
|  |  |  | Stage 1: All Non-Obese Patients | | | Stage 2: All Obese Patients | | | Combined | | |
| Affymetrix  Probeset | Gene | Chrom | Fold Change in Tumor Tissue  (p-value) | Fold Change in Normal Tissue  (p-value) | Interaction  p-value | Fold Change in Tumor Tissue  (p-value) | Fold Change in Normal Tissue  (p-value) | Interaction  p-value | Fold Change in Tumor Tissue  (p-value) | Fold Change in Normal Tissue  (p-value) | Interaction p-value |
| **240292_x_at** | **ANKS1B** | **12** | **0.92 (0.013)** | **1.08 (0.02)** | **0.00082** | **0.98 (0.64)** | **1.11 (0.005)** | **0.018** | **0.94 (0.014)** | **1.09 (0.0013)** | **0.00005** |
| 227440_at |  |  | 0.99 (0.89) | 1.25 (0.0063) | 0.022 | 0.81 (0.018) | 1.04 (0.64) | 0.026 | 0.93 (0.23) | 1.18 (0.0076) | 0.002 |
| 227441_s_at |  |  | 0.88 (0.21) | 1.12 (0.24) | 0.074 | 0.6 (0.0006) | 0.88 (0.33) | 0.015 | 0.79 (0.0033) | 1.04 (0.59) | 0.0069 |
| 227439_at |  |  | 0.88 (0.14) | 1.05 (0.56) | 0.11 | 0.88 (0.18) | 1.12 (0.23) | 0.077 | 0.88 (0.055) | 1.07 (0.31) | 0.022 |
| 219989_s_at |  |  | 1.0 (0.91) | 1.01 (0.84) | 0.95 | 1.01 (0.8) | 0.97 (0.49) | 0.51 | 1.0 (0.84) | 1.0 (0.89) | 0.81 |
| **241949_at** | **ACOT6** | **14** | **0.88 (0.0098)** | **1.07 (0.15)** | **0.00085** | **0.89 (0.1)** | **1.04 (0.56)** | **0.034** | **0.88 (0.0019)** | **1.06 (0.13)** | **0.00006** |
| **236999_at** | **PPWD1** | **5** | **1.13 (0.0062)** | **0.99 (0.74)** | **0.0015** | **1.2 (0.0034)** | **1.04 (0.5)** | **0.038** | **1.14 (0.00023)** | **1.0 (0.99)** | **0.00016** |
| 213483_at |  |  | 1.23 (0.044) | 0.94 (0.56) | 0.05 | 1.1 (0.45) | 1.0 (1.0) | 0.46 | 1.19 (0.03) | 0.96 (0.62) | 0.035 |
| **233996_x_at** | **EYS** | **6** | **0.94 (0.022)** | **1.03 (0.27)** | **0.0022** | **1.0 (1)** | **1.06 (0.045)** | **0.046** | **0.96 (0.033)** | **1.04 (0.077)** | **0.00026** |
| **241459_at** | **LIMCH1** | **4** | **1.27 (0.06)** | **0.89 (0.37)** | **0.0077** | **1.9 (0.0032)** | **1.09 (0.65)** | **0.02** | **1.43 (0.0011)** | **0.95 (0.62)** | **0.00047** |
| 212328_at |  |  | 1.15 (0.36) | 0.77 (0.093) | 0.042 | 1.0 (1.0) | 0.68 (0.11) | 0.093 | 1.11 (0.42) | 0.75 (0.033) | 0.01 |
| 212325_at |  |  | 1.22 (0.19) | 0.96 (0.77) | 0.11 | 1.06 (0.77) | 0.82 (0.29) | 0.24 | 1.18 (0.19) | 0.91 (0.47) | 0.043 |
| 212327_at |  |  | 1.15 (0.3) | 0.92 (0.55) | 0.2 | 1.35 (0.13) | 0.91 (0.62) | 0.061 | 1.21 (0.086) | 0.92 (0.48) | 0.046 |
| 232457_at |  |  | 1.09 (0.098) | 1.12 (0.029) | 0.51 | 1.29 (0.00028) | 1.32 (0.00013) | 0.7 | 1.15 (0.0016) | 1.18 (0.00018) | 0.44 |
| **207568_at** | **CHRNA6** | **8** | **0.92 (0.24)** | **1.15 (0.064)** | **0.0075** | **0.95 (0.62)** | **1.23 (0.034)** | **0.037** | **0.93 (0.2)** | **1.17 (0.0069)** | **0.00058** |
| **210472_at** | **MT1G** | **16** | **1.0 (0.97)** | **1.47 (0.00013)** | **0.0045** | **0.93 (0.62)** | **1.4 (0.032)** | **0.018** | **0.98 (0.82)** | **1.45 (0.0000099)** | **0.00061** |
| 204745_x_at |  |  | 1.37 (0.27) | 1.51 (0.17) | 0.81 | 1.21 (0.64) | 1.09 (0.84) | 0.83 | 1.33 (0.22) | 1.37 (0.19) | 0.93 |
| **242463_x_at** | **ZNF600** | **19** | **1.48 (0.0064)** | **0.98 (0.87)** | **0.0077** | **1.78 (0.001)** | **1.31 (0.083)** | **0.045** | **1.56 (0.00013)** | **1.07 (0.53)** | **0.0012** |
| **210322_x_at** | **UTY** | **Y** | **0.98 (0.76)** | **1.13 (0.079)** | **0.0086** | **0.96 (0.72)** | **1.22 (0.075)** | **0.0093** | **0.98 (0.68)** | **1.16 (0.012)** | **0.00029** |
| **1557478_at** | **NA** | **NA** | **1.22 (0.03)** | **0.95 (0.55)** | **0.00052** | **1.88 (0.00002)** | **1.31 (0.022)** | **0.0011** | **1.38 (0.00012)** | **1.04 (0.62)** | **0.000002** |
| **1558410_s_at** | **NA** | **NA** | **1.42 (0.013)** | **0.93 (0.58)** | **0.0049** | **2.49 (0.0000087)** | **1.63 (0.0038)** | **0.017** | **1.66 (0.000044)** | **1.1 (0.44)** | **0.0006** |
| **210717_at** | **NA** | **NA** | **1.74 (0.00062)** | **0.89 (0.47)** | **0.0033** | **2.12 (0.00026)** | **1.41 (0.053)** | **0.027** | **1.83 (0.000011)** | **1.03 (0.8)** | **0.00086** |
| **232324_x_at** | **NA** | **NA** | **1.17 (0.004)** | **0.97 (0.52)** | **0.0081** | **1.27 (0.0018)** | **1.03 (0.61)** | **0.039** | **1.2 (0.000058)** | **0.99 (0.77)** | **0.0011** |
| **232369_at** | **NA** | **NA** | **1.36 (0.021)** | **0.89 (0.4)** | **0.0081** | **2.07 (0.00065)** | **1.3 (0.15)** | **0.031** | **1.53 (0.00016)** | **1.0 (0.99)** | **0.00078** |
| **244290_at** | **NA** | **NA** | **1.32 (0.0038)** | **0.99 (0.94)** | **0.0015** | **1.66 (0.000041)** | **1.26 (0.022)** | **0.033** | **1.4 (0.000035)** | **1.06 (0.42)** | **0.00019** |

Chrom denotes chromosome.

Interaction p-value denotes the smoking status-by-tissue type interaction p-value.

NA denotes that there is no gene annotation available for the corresponding Affymetrix probeset.
